# The recombinant plant *Bauhinia bauhinioides* elastase inhibitor reduces rat thrombus without alterations in hemostatic parameters

**DOI:** 10.1038/s41598-021-92745-4

**Published:** 2021-06-29

**Authors:** Cleide Oliveira, Mayara Vioto Valois, Tatiana Fontes Ottaiano, Antonio Miranda, Daiane Hansen, Misako Uemura Sampaio, Maria Luiza Vilela Oliva, Francisco Humberto de Abreu Maffei

**Affiliations:** 1 Departamento de Bioquímica, Universidade Federal de São Paulo, Rua Três de Maio, 100, São Paulo, SP 04044-020 Brazil; 2 Departamento de Biofísica, Universidade Federal de São Paulo, São Paulo, SP 04044-020 Brazil; 3 Departamento de Cirurgia e Ortopedia, Universidade Estadual Paulista, Botucatu, SP 18618-970 Brazil

**Keywords:** Biochemistry, Drug discovery, Diseases, Drug development

## Abstract

The anti-inflammatory effects of the plant protease inhibitor BbCI (*Bauhinia bauhinioides* cruzipain inhibitor), which blocks elastase, cathepsin G, and L, and proteinase 3 has been demonstrated. Here, we investigated the recombinant rBbCI-His_(6)_ (containing a histidine tail) in an experimental venous thrombosis model of vena cava (VC) ligature in rats, comparing to heparin. We evaluate the effects of the inhibitors (native or recombinant) or heparin on the activated partial thromboplastin time (aPTT) and prothrombin time (PT) in human and rat plasmas. The rats undergoing treatment received a saline solution or increasing concentrations of rBbCI-His_(6)_, heparin, or a mixture of both. After 4 h of ligature VC, thrombus, if present was removed and weighed. aPTT, PT, and cytokines were measured in blood collected by cardiac puncture. aPTT, PT, and bleeding time (BT) were also measured at the time of VC (vena cava) ligature. rBbCI-His_(6)_ (0.45 or 1.40 mg/kg) does not alter aPTT, PT or BT. No differences in coagulation parameters were detected in rBbCI-His_(6)_ treated rats at the time of VC ligature or when the thrombus was removed. There was a significant decrease in the weight of thrombus in the animals of the groups treated with the rBbCI-His_(6)_ (1.40 mg/kg), with the rBbCI-His_(6)_ mixture (1.40 mg/kg) + heparin (50 IU/kg) and heparin (100 IU/kg) in relation to control group (saline). The growth-related oncogene/keratinocyte chemoattractant (GRO/KC) serum levels in rats treated with rBbCI-His_(6)_ (1.40 mg/kg) or heparin (200 IU/kg) were reduced. In the experimental model used, rBbCI-His_(6)_ alone had an antithrombotic effect, not altering blood clotting or bleeding time.

## Introduction

Deep venous thrombosis (DVT) and its main complication, pulmonary thromboembolism, jointly with venous thromboembolism (VTE), constitute a global disease that is the third cause of cardiovascular deaths^1,2^.

DVT can occur without a provoking cause, and it is mainly due to genetic or acquired thrombophilia^3,4^. The occurrence of DVT, a major risk of perioperative sudden death, is associated with specific surgical procedures, including those for orthopaedic major joint surgeries. However, most cases present a triggering transient factor, like surgery with general anesthesia for more than 30 min, bed rest for more than three days at the hospital or at home with an acute disease, trauma vascular injury, estrogen therapy, pregnancy or puerperium, leg injury associated with reduced mobility or provoked by a persistent risk factor such as active cancer, inflammatory bowel disease, among others^5,6^.

Since the 1960s, it is known that this flow reduction causes the formation of vortices at the level of valve sinus in leg veins. These events could lead to an accumulation of platelets that could be responsible for local activation of coagulation and accumulation of activated clotting factors, which would lead to thrombus formation and extension^7^. Also, the presence of leukocytes in the site was reported^8,9^; however, no special attention was given to this presence.

More recently, several experimental studies showed the importance of leukocytes in the formation of venous thrombus^10,11^. Leukocytes, mainly monocytes, would be attracted, activated, and adhered to the endothelial surface with a possible migration to adjacent regions, according to a gradient of substances released or expressed by the endothelium ischemia caused by a decrease in blood flow, to trauma, or decreased blood flow. Activated monocytes and endothelial cells would then release/express a tissue factor (TF) that would result in the activation of blood coagulation, promoting the adhesion and activation of platelets^12,13^. In addition to participating in this sequence of events that lead to the formation of the thrombus, these cells along with the macrophages also participate in fibrinolysis and resolution of the thrombus^14,15^. Inflammation can be important in both arterial and venous thrombosis, being a possible common mechanism by which different risk factors can initiate the formation of thrombi in arteries and veins^16^.

Anticoagulants (heparin, low molecular heparins, fondaparinux, anti-vitamin K, and more recently direct oral anticoagulants) are used in the prophylaxis and treatment of VTE. Although efficient, they may present bleeding complications that can increase the morbidity and mortality of patients^17,18^.

The most known pharmacological action of heparin is the inactivation of coagulation factors due to their interaction with antithrombin. However, from the knowledge that unfractionated heparin and low molecular weight heparin have anti-inflammatory activity, the role of heparin(s) in the prophylaxis of venous thrombosis is currently related to both anticoagulant and anti-inflammatory effects due to its ability to inhibit the leukocytes adhesion, migration, and neutrophil activation blocking enzymes released, proinflammatory cytokines production and degradation or formation of neutrophil extracellular traps (NETs)^19–21^.

Human neutrophils store mainly pro-inflammatory enzymes, such as elastase (HNE), cathepsin G (CG), and proteinase 3 (PR3), in their azurophil granules^22^. These enzymes are capable of generating various physiological responses due to their different biological activities on endogenous and exogenous substrates; they need to be strictly regulated to avoid uncontrolled proteolysis and/or inflammation in the body^23^. When and if necessary, the neutralization of these enzymes occurs by endogenous inhibitors of serine proteases that constitute and maintain the balance between proteolysis and anti-proteolysis^24–26^.

In 2001, Oliveira et al.^27^ isolated a protein from the seeds of *Bauhinia bauhinioides* named BbCI that is capable of inhibiting the "in vitro" activity of serine proteases such as HNE, CG, and PR3 and does not interfere with the activities of factors IIa and Xa of coagulation. In in vivo models of acute inflammation^28^, BbCI demonstrated effectiveness in reducing cell adhesion, migration, and chemotaxis by decreasing the amount of cytokine-induced neutrophil chemoattractant-1 (CINC-1), local and systemic. Considering the anti-inflammatory action of BbCI by inhibiting the pro-inflammatory enzymes HNE and CG, and its ineffectiveness on factors IIa and Xa activities, we proposed to verify the antithrombotic properties with a possible less hemorrhagic effect in the VC ligature model in rats.

## Material and methods

### Reagents

Human neutrophil elastase (HNE) (EC.3.4.21.37), human neutrophil proteinase 3 (PR3) (EC.3.4.21.76), and human neutrophil cathepsin G (CG) (EC.3.4.21.20) were acquired from Calbiochem Ltd. (San Diego, USA). The unfractionated heparin (Hepamax-S^®^, Porcine) was obtained from Blau Pharmaceutical (Cotia, SP, Brazil). MeO-Suc-Ala-Ala-Pro-Val-pNan, MeO-Suc-Ala-Ala-Pro-Phe-pNan, and MeO-Suc-Ala-Ala-Pro-Val-pNan were acquired from Calbiochem Ltd. (San Diego, USA). The PT and aPTT reagents (Thromborel S and Dade Actin Activated Cephaloplastin reagent) were obtained from Dade Behring (Marburg, Germany). All other used reagents were of analytical grade.

### Elastase inhibitors

#### Native inhibitor

The BbCI (*Bauhinia bauhinioides* cruzipain inhibitor) was extracted and purified from *B. bauhinioides* seeds, as previously described by Oliveira et al.^27^.

#### Recombinant inhibitor

The recombinants inhibitors, rBbCI or rBbCI-His_(6)_, were obtained by heterogonous expression in *E. coli* (pET28a plasmid) and purified and characterized according to its activity on HNE^29^. Part of the fraction containing the 6xHis was pooled and frozen for further experiments including the thrombosis experimental model. The remaining protein was dialyzed against buffer (20 mM Tris–HCl, pH 8.4, at 25 °C, 150 mM NaCl, and 2.5 mM CaCl_2_) and cleaved by the addition of bovine thrombin (0.5 U/mg of protein)**.** For separation of thrombin and other contaminants, the material was subjected to molecular exclusion chromatography on Superdex 75 10/300 GL column (Amersham Biosciences, GE Healthcare, Amersham, UK) using ÄKTA system (GE Healthcare, New Jersey, USA), equilibrated with 0.1 M Tris–HCl buffer, 8.0, under a constant flow of 0.5 mL/min.

**Figure 1:**
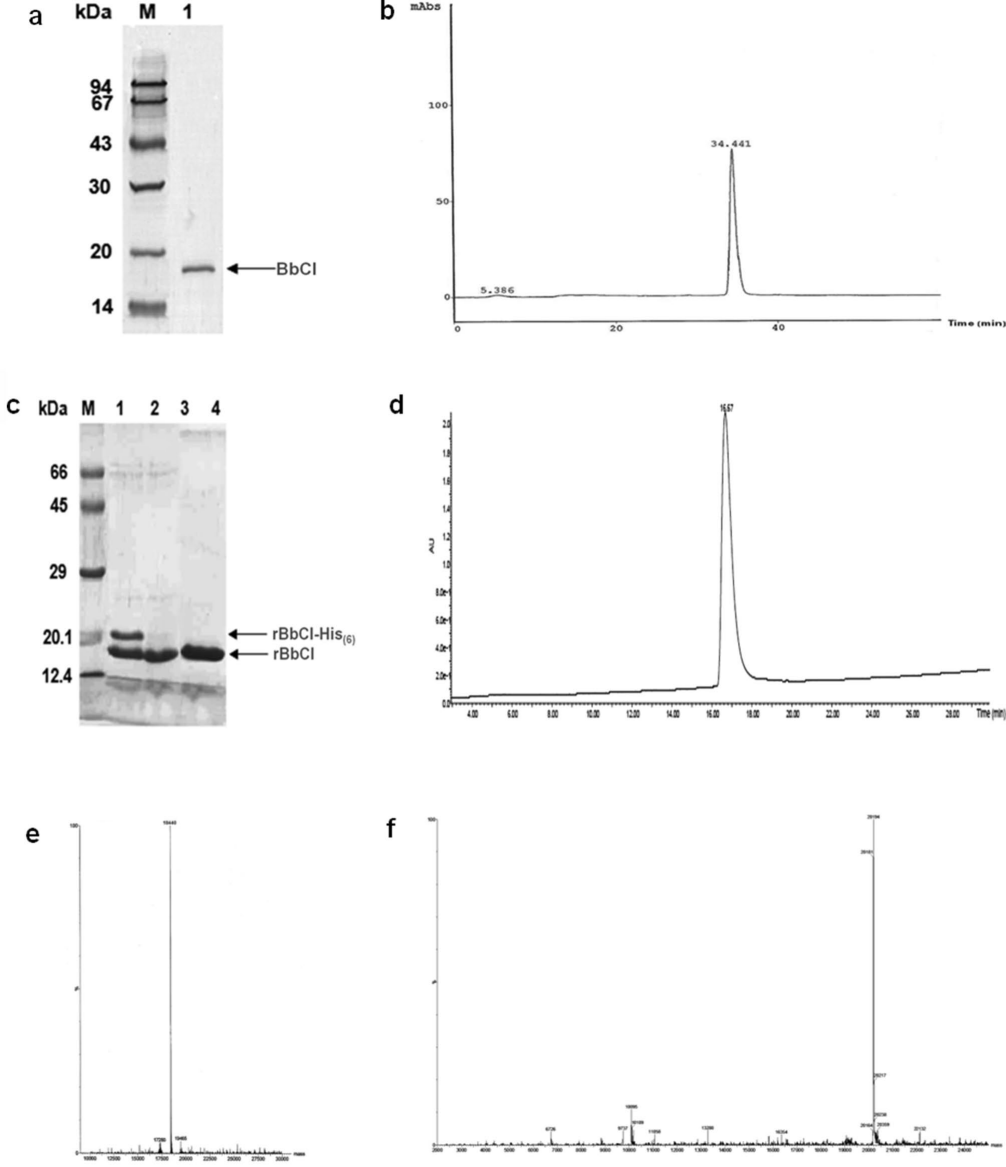
Purity analyses of the inhibitors. SDS-PAGE (15%) stained with Coomassie-blue. **(a)** BbCI after reverse phase chromatography and under reducing conditions. (M) Molecular mass standard. (1) BbCI (30 µg). **(c)** rBbCI after the cleavage of the fusion peptide. M) Molecular mass standards. rBbCI subjected to cleavage with (1) 0.5 IU of thrombin after 1 h of incubation at 18 °C. (2) 0.5 IU of thrombin after 4 h of incubation at 18 °C. 3) rBbCI after thrombin cleavage (4 h) and gel filtrated in Superdex 75 equilibrated with 0.1 M Tris–HCl buffer (pH 8.0). The gels were scanned (HP Photosmart C4280) to obtain the images which are shown in black and white. The resize was realized to facilitate the comparison between protein migrations and the specific area of the image **(c)** was cropped using the GIMP (GNU Image Manipulation Program) Portable, image editor. The gels are presented in a Supplementary Information file. Reverse-phase chromatography. HPLC system, C18 protein/peptide column (15 cm × 4.6 mm; Vydac) equilibrated with 0.1% TFA in water, and elution with 90% acetonitrile gradient (0 to 90%) **(b)** BbCI and acetonitrile gradient (5 to 95%) **(d)** rBbCI. Chromatography performance at room temperature and flow rate of 0.7 mL/min. LC/MS analysis. Mass spectrometer, 3100 model coupled with a system Alliance model 2690, Waters Nova-Pak C18 column (2.2 × 150 mm, 3.5 µm particle size, 60 A° pore size. The solvents used were: A: 0.1% TFA/H_2_O and B: 90% CH3CN/A; with a gradient: 5–95% B for 30 min at 250 µL/min, λ: 214 nm and mass range: 200–2000 m/z **(e)** rBbCI and **(f)** rBbCI-His_(6)_.

The SDS-PAGE of the proteins showed the presence of a single major peak and the purity was also confirmed by reverse phase chromatography and mass spectrometry (Fig. 1).

### Electrospray mass spectrometry characterization

The samples were analyzed by LC/MS to determine their molecular weight and purity using a mass spectrometer, model 3100, coupled with a system Alliance model 2690, both from Waters (Milford, USA/MA). A Waters Nova-Pak C18 column (2.2 × 150 mm, 3.5 µm particle size, and 60 A° pore size (Waters Corporations; Milford, USA/MA) was employed. The following solvents were used: A) 0.1% TFA/H_2_O and B) 90% CH_3_CN/A; with the gradient from 5 to 95% B for 30 min at 250 µL/min; λ at 214 nm; and mass range of 200–2000 m/z.

### Inhibitory activity determination

HNE (26 nM), CG (0.5 µM), and PR3 (81 nM) were pre-incubated with increasing concentrations of inhibitors or heparin in 0.1 mM Tris–HCl buffer, pH 7.5, containing 0.5 M NaCl, for 10 min in a volume of 230 µL. After this time, substrates were added (20 µL), and the residual activity was measured with (1 mM) MeO-Suc-Ala-Ala-Pro-Val-pNan, (1 mM) MeO-Suc-Ala-Ala-Pro-Phe-pNan, and (2 mM) MeO-Suc-Ala-Ala-Pro-Val-pNan, respectively. The release of pNA was monitored at 405 nm for 30 min. The assay was mandated at 37 °C. The assays were performed as previously described^27^. Each assay was performed in triplicate.

### Animals

Male Wistar rats (280–380 g), obtained from the Institute of Pharmacology and Molecular Biology Professor Dr. Ribeiro do Vale at the Federal University of São Paulo (UNIFESP), were used in this study. Animals had free access to water, and the diet was reduced by 50% at 12 h before the beginning of the experiment. Animals were acclimated in the experimental room at least 1 h before the experiment starts; they were maintained at 25 °C after surgery and until the end of the experiment (for 4 h during this period they had free access to water and food). All animal care and experimental procedures were approved by the Research Ethics Committee of the Federal University of São Paulo, São Paulo, Brazil (CEUA No 1793/11), and all methods were carried out in accordance with the ARRIVE guidelines.

### Experimental groups used to study the antithrombotic effect

The animal experiments were formed by ligature group (group to investigate the antithrombotic effects of the substances), and controls: sham surgery and substances control after a half-hour (control of substance action after ½ h of treatment, moment of ligature), and substances control group (control of the action of the substance after 4½ h of treatment).

In the ligature group, the rats were anesthetized, treated with the substances (9 subgroups), and after 30 min submitted to IVC ligature. After 4 h the rats were re-anesthetized, when the thrombus was present, it was collected and weighed on an analytical balance, and blood was collected to further aPTT, PT, and cytokines determination.

**Figure 2:**
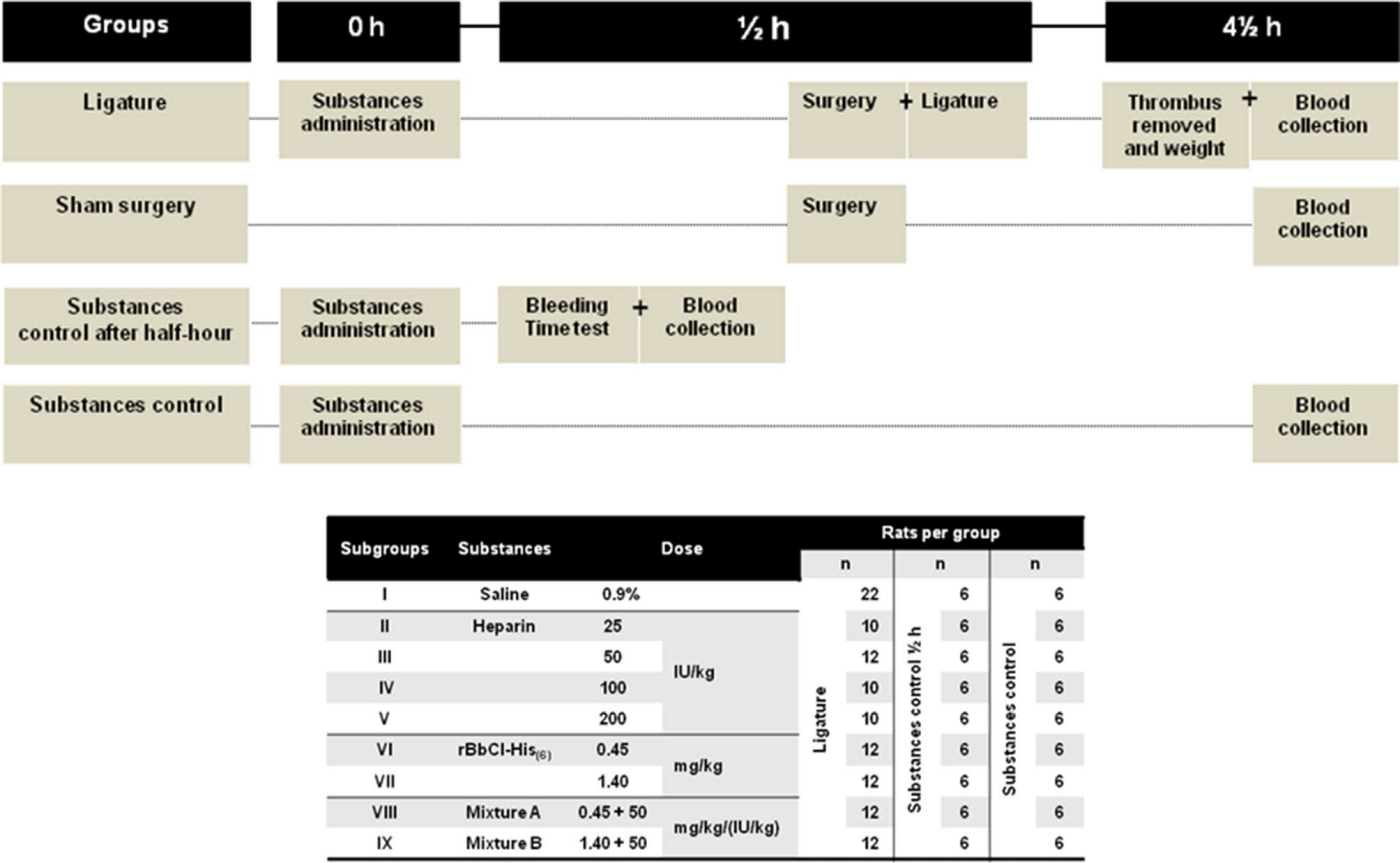
Timeline of the procedures realized indicating the experimental groups. Rats were allocated to 4 groups: Ligature group (treated with the studied substances, and submitted to IVC ligature), subdivided into 9 groups; Sham surgery (no treated, and submitted to surgery without VC ligature, n = 20); Substances control group after a half-hour (treated with the substances, non-operated, non-ligated), and substances control group after 4½ h treatment (treated with the studied substances, non-operated, non-ligated). The two groups were subdivided into 9 groups.

In groups sham surgery (anesthetized, no treated, and submitted to surgery without VC ligature) and substances control (anesthetized, treated with the studied substances, non-operated, non-ligated) 4 ½ hours later the animals were re-anesthetized, blood was collected and stored to test for aPTT, PT, and cytokines. To assess whether the substances have acted at the time of ligature a control group of substances a half-hour after treatment was monitored. The rats were anesthetized and treated with the substances under study (subdivided into nine subgroups, non-operated, non-ligated). Differently from the other groups, after 30 min of the administration of the substances, the BT was determinate in the vein of the tail and then the blood was collected and stored for aPTT and PT determination. Rats from all groups after blood collection were immediately euthanized (Fig. 2).

### Induction of thrombosis

Animals were anesthetized with ketamine (40 mg/kg) and xylazine (10 mg/kg) via intramuscular injection and treated through the tail vein with 300 µL of the studied substances.

The IVC thrombosis was induced as proposed by Reyers et al.^30^ with small modifications. Briefly, the abdomen was opened by a median incision, and the gut exteriorized and maintained in a small plastic bag containing 1.0 mL of Ringer lactate solution at 25 °C*.* The VC was dissected free, and the tributary veins, if present between the left renal and iliac veins, were ligated. The VC was ligated immediately distal to the left renal vein.

The abdominal incision was closed with a continuous suture with mono nylon 4–0. Four hours later, the animals were re-anesthetized, the abdominal incision was reopened, and the segment of vena cava between the renal and iliac veins was removed and opened longitudinally; if a thrombus was present, it was removed and placed on a piece of filter paper of known weight and weighed immediately on an analytical balance.

Immediately after this procedure, blood was withdrawn by cardiac puncture, and an overdose of anesthetics euthanized the animals. The serum and plasma were separated and frozen for later measurement of aPTT, PT, and cytokines.

### Obtaining serum and plasma

Plasma samples of rats and humans were collected in tubes containing 3.8% (w/v) trisodium citrate solution, and serum was obtained by blood collection without anticoagulant. The platelet-poor plasma (PPP) and serum samples were separated by centrifugation at 2500x*g* for 20 min at 4 °C. Samples of human plasma were collected by peripheral venipuncture from ten healthy and unmedicated male volunteers between 20 and 30 years old. Rats’ serum and plasma samples were obtained by cardiac puncture in anesthetized animals. An overdose of anesthetics immediately euthanized animals after the collection of blood samples was completed. Serum or plasma samples were fractionated and stored at − 70 °C.

### Hemostasis studies

1. aPTT and PT were determined in rat PPP (platelet-poor plasma) in all experimental groups, regardless of the time when blood was collected;

2. aPTT and PT were determined in human or rat PPP to which the increasing concentrations of the studied substances were added: heparin, BbCI, rBbCI, rBbCI-His_(6)_, and rBbCI-His_(6)_ associated with heparin (mixture A, B, C, D) and saline (Table 3); the final volume was completed with saline. The clotting time was measured in seconds according to the technique described by Silva et al.^31^.

#### Ex-vivo

Plasma samples were collected from rats at two different times: at the time of ligature (substances control group after a half-hour) and 4 h after the ligature (in the ligature groups, simulated surgery, and in the substances control group). The aPTT and PT were determined using platelet-poor plasma (PPP) of rats treated with the studied substances (Table 2). The clotting time was measured in seconds, following the technique described above.

### Bleeding time (BT)

A partial 4 mm transverse incision was made 2 cm from the tail tip. Tails were immediately immersed in saline at 37 °C, and the BT was measured as described by Perzborn, et al*.*^32^.

### Serum cytokine determination in the experimental group

The concentrations of cytokines were determined in rat serum samples stored at − 80 °C. At the moment of analysis, serum was centrifuged for 10 min at 8000×*g* at 25 °C. Tumor necrosis factor (TNF-α), interleukins (IL-1α, IL-1β, IL-6), chemokine monocyte chemoattractant protein 1 (MCP-1), macrophage inflammatory protein-1alpha (MIP-1α), and GRO/KC were determined using a rat cytokine/chemokine magnetic bead panel (Milliplex MAP kit, Millipore Corp., Billerica, MA, USA). Results were measured in a Luminex® 200TM System (xponent 3.1 software, Luminex Corp, Austin, Texas, USA), according to the manufacturer’s instruction.

### Statistical analysis

Data were expressed as mean ± s.d. and analyzed in the GraphPad Prism 5 Software for Windows (GraphPad Software, San Diego, CA, USA). A statistically significant difference was determined by the Kruskal–Wallis test. Dunn's multiple comparisons test was used if data were not normally distributed. Significant differences were considered when *p* ≤ 0.05.

## Results

### Inhibitory properties of native and recombinant proteins

The biological activity of the recombinant BbCl inhibitor (with and without the histidine tail) was compared to that of the native protein on different serine proteases, including those of neutrophils^29^. The native BbCI blocks the activity of neutrophil elastase, cathepsin G, and proteinase 3. The recombinant rBbCI (without histidine tail) or rBbCI-His_(6)_ (with histidine tail) showed similar inhibition constants compared to the native protein (BbCl), except for the inhibition of PR3 that is achieved only by the native protein. On the other hand, the efficacy of rBbCl-His_(6)_ in inhibiting cathepsin G is 8.0 and 10.5 times better compared to the native protein BbCI and the recombinant rBbCI respectively, although only the 10.5 times increase showed a significant difference (Table 1). In addition to the neutrophilic enzymes, some clotting enzymes such as plasma kallikrein, Xa, and IIa were tested against the inhibitors. However, as has already been demonstrated by Oliveira et al.^27^ and Araujo et al.^29^ for BbCI and rBbCI, respectively, none of them, including rBbCI-His_(6)_ inhibit these enzymes.

**Table 1:** Studies of the inhibitory action of BbCI and its recombinant form on neutrophil enzymes.

Neutrophil serine proteases	Inhibitors (K_*iapp*_ nM)		
	BbCI	rBbCI	rBbCI-His_(6)_
Human cathepsin G—CG	160.0	210.0	20.0*
Human elastase neutrophil—HNE	5.3	1.7	2.1
Human proteinase 3—PR3	7.0	No inhibition	No inhibition

Data are expressed as mean and *p˂0.05, versus the corresponding Kiapp values of rBbCI was considered significant.

Enzymes activities of CG (0.5 µM), HNE (26 nM), and PR3 (81 nM) were determined by pre-incubation with increasing concentrations of the different inhibitors in 0.1 mM Tris–HCl buffer, pH 7.5 containing 0.5 M NaCl, 37 °C for 10 min. Residual activity was measured with (1 mM) MeO-Suc-Ala-Ala-Pro-Phe-pNan, (1 mM) MeO-Suc-Ala-Ala-Pro-Val-pNan, and (2 mM) MeO-Suc-Ala-Ala-Pro-Val-pNan, respectively. *Kiapp (apparent inhibitory constant)*; BbCI (*Bauhinia bauhinioides* cruzipain inhibitor), rBbCI (recombinant BbCI without histidine tail), rBbCI-His_(6)_ (recombinant BbCI containing the histidine tail). Data are expressed as mean and *p˂0.05, versus the corresponding Kiapp values of rBbCI was considered significant.

### Comparison of the anticoagulant effect of BbCl, rBbCI, and rBbCI-His_(6)_ to heparin on coagulation parameters (aPTT and PT) in human and rat plasma

To compare the effects of BbCI, rBbCI, rBbCI-His_(6)_ to heparin on coagulation we evaluate “in vitro" using human and rat plasma, the influence on aPTT and PT at the same doses administered "in vivo”. BbCI and rBbCI, at the dose of 1.4 mg/kg, prolonged the aPTT of human plasma by 1.8 and 2.2 times, respectively, and 1.3 and 1.5 times of rat plasma, compared to the control group. A subtle extension in PT in rats with the use of rBbCI (1.4 mg/kg) was observed; 0.45 mg/kg rBbCI only prolonged 1.5 times the aPTT in human plasma. The rBbCI-His_(6)_ (0.45 and 1.4 mg/kg) did not affect PT or aPTT. The slight increase in PT was not significant confirming that BbCI and its recombinant forms do not interfere with the extrinsic pathway enzyme activities. Plasma treated with heparin in all doses was non-coagulable (> 300 s) at both parameters, aPTT, and PT. The association of rBbCI-His_(6)_ and heparin (mixture A, B, C, and D) did not change the results found when using heparin alone (Table 2).

**Table 2:** Determination of the anticoagulant action of heparin and BbCI (aPTT and PT) in human and rat plasma.

Groups		Human plasma		Rat plasma	
		aPTT (s)	PT (s)	aPTT (s)	PT (s)
**Saline**		21.36 ± 1.82	16.10 ± 0.60	27.90 ± 1.14	15.10 ± 0.57
**Heparin (IU/kg)**					
25		> 300	> 300	> 300	> 300
50		> 300	> 300	> 300	> 300
100		> 300	> 300	> 300	> 300
200		> 300	> 300	> 300	> 300
**BbCI (mg/kg)**					
0.45		24.73 ± 2.72	16.90 ± 0.95	22.38 ± 1.37	15.62 ± 0.48
1.40		39.14 ± 5.05***	16.90 ± 0.86	36.10 ± 1.94**	16.61 ± 0.58
**rBbCI (mg/kg)**					
0.45		32.71 ± 2.72***	18.40 ± 1.86	26.78 ± 1.24	14.63 ± 0.53
1.40		46.50 ± 3.83***	18.10 ± 1.21	42.96 ± 2.15***	17.31 ± 1.30*
**rBbCI-His**_**(6)**_** (mg/kg)**					
0.45		26.70 ± 0.47	15.50 ± 0.40	27.07 ± 2.70	15.33 ± 1.20
1.40		23.80 ± 0.86	15.30 ± 1.21	27.63 ± 0.87	15.06 ± 0.82
**rBbCI-His**_**(6)**_** (mg/kg) + heparin (IU/kg)**					
Mixture A	0.45 + 50	> 300	> 300	> 300	> 300
Mixture B	1.40 + 50	> 300	> 300	> 300	> 300
Mixture C	0.45 + 100	> 300	> 300	> 300	> 300
Mixture D	1.40 + 100	> 300	> 300	> 300	> 300

aPTT or PT parameters were determined using a plasma pool of 10 rats and a plasma pool of healthy human volunteers (n = 10). The plasmas were tested with saline or heparin or inhibitors or mixtures. Data are expressed as mean ± s.d. *p˂0.05, **p˂0.01, ***p˂0.001 versus corresponding values of plasma tested with saline. The normal range of the values of the PT (14.6 ± 1.7 s) and aPTT (26.6 ± 3.6 s) of the rats used in our laboratory was determined using Dade reagents. aPTT (activated partial thromboplastin time), PT (prothrombin time), Heparin (unfractionated heparin).

### The effects of heparin and rBbCI-His_(6)_ at the time of rat VC ligature, and 4 h after ligature in the BT, aPTT, and PT parameters

At the time of VC ligature, the BT was prolonged in 23% and 35% in animals treated with heparin at 50 and 100 IU/kg, respectively, and compared to the control group. This result was different from those observed at the dose of 200 IU/kg in which the BT extended in approximately 295% (3 times, Table 3). Conversely, rBbCI-His_(6)_ (0.45 or 1.4 mg/kg) did not alter the BT at the time of VC ligature. Interestingly, the BT from the association inhibitor with heparin (mixture A and B) was similar to that observed using heparin alone at the dose of 50 IU/kg (Table 3). Regarding coagulation parameters, the aPTT of animals treated with heparin at 50 or 100 IU/kg was prolonged 2 and 7 times, respectively. The highest dose of 200 IU/kg heparin produced non-coagulable plasma (> 300 s, Table 3).

**Table 3:** Effect of substances (heparin and BbCl) at the time of ligature and after 4 h (ex-vivo) in the BT, aPTT, and PT parameters.

Experimental Groups		30 min (ligature time)		4 h (after ligature)	
		BT(s)	aPTT(s)	aPTT(s)	PT(s)
Saline		87.17 ± 8.70	29.90 ± 3.56	20.18 ± 1.77	13.70 ± 1.30
**Heparin (IU/kg)**					
25		85.67 ± 9.11	34.43 ± 1.72	17.48 ± 1.12	11.90 ± 0.72
50		107.30 ± 16.57	59.98 ± 5.30**	18.49 ± 1.56	12.90 ± 0.45
100		117.50 ± 15.65*	221.30 ± 32.32 ***	18.95 ± 1.55	12.00 ± 0.74
200		257.30 ± 26.41***	> 300	23.14 ± 2.40	12.95 ± 0.40
**rBbCI-His**_**(6)**_** (mg/kg)**					
0.45		85.17 ± 5.11	31.78 ± 3.60	19.61 ± 1.95	13.50 ± 0.86
1.40		80.10 ± 5.62	28.10 ± 1.64	19.50 ± 1.37	12.50 ± 1.05
**rBbCI-His**_**(6)**_** (mg/kg) + heparin (IU/kg)**					
Mixture A	0.45 + 50	105.80 ± 9.95	61.00 ± 2.25***	20.70 ± 1.21	13.60 ± 0.75
Mixture B	1.40 + 50	98.33 ± 12.31	55.60 ± 5.05*	21.66 ± 1.05	15.20 ± 1.06

BT and aPTT were determinate in tail and plasma respectively in rats treated intravenously with saline or heparin or rBbCI-His_(6)_ or mixture, 30 min before the ligature vena cava (substances control group after a half-hour of treatment).

aPTT and PT were realized in rat plasma after 4 h of the ligature (group *ex-vivo*). Values are presented as mean ± s.d.. *p˂0.05, **p˂0.01, ***p˂0.001 versus corresponding values of plasma tested with saline. BT- bleeding time.

It is worth mentioning that the treatment with rBbCl-His_(6)_ (0.45 or 1.4 mg/kg) did not alter the BT and aPTT at the ligature time (Table 3). In the A or B mixture, the aPTT was prolonged only by 2 times, similar to that using heparin alone at the dose of 50 IU/kg. After 4 h all groups showed aPTT and PT values similar to those in the control group (Table 3).

### Influence of heparin or rBbCl-His_(6)_ on the enzymatic activity of neutrophil serine proteases

**Figure 3:**
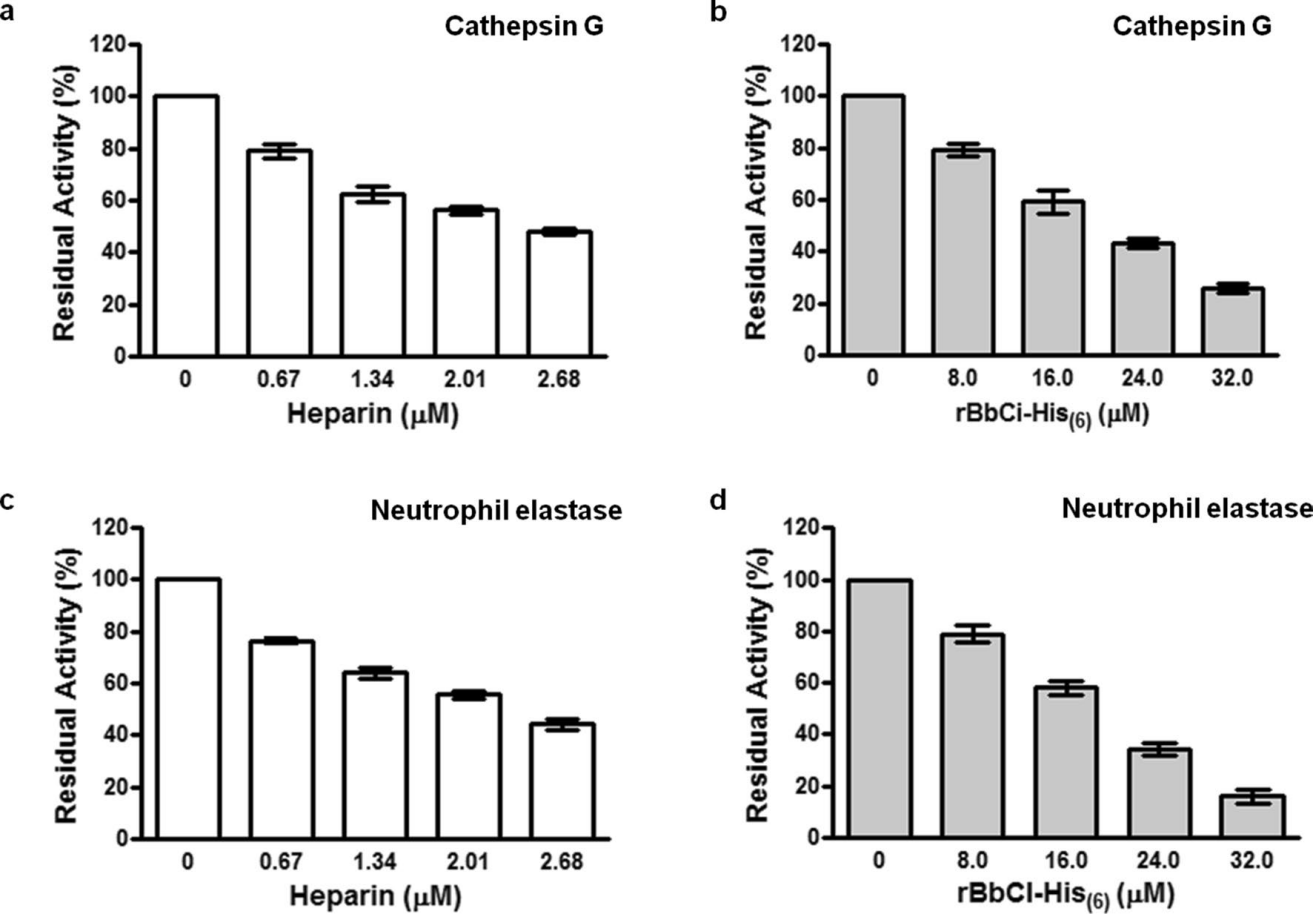
Influence of heparin or rBbCl-His_(6)_ on the enzymatic activity of neutrophil serine proteases. Cathepsin G (0.5 µM) **(a**,**b)** or elastase neutrophil (26 nM) **(c**,**d)** were pre-incubated (10 min, at 37 °C, 0.1 mM Tris–HCl buffer at pH 7.5 containing 0.5 M NaCl) in the presence of increasing concentrations of heparin (unfractionated heparin) or rBbCI-His_(6)_, incubated for 30 min at 37 °C. The residual activity of each enzyme was measured using MeO-Suc-Ala-Ala-Pro-Phe-pNan and MeO-Suc-Ala-Ala-Pro-Val-pNan (1 mM) for cathepsin G and elastase, respectively.

The effects of heparin and inhibitor were compared in the alteration of the cathepsin G and elastase catalytic action from human neutrophils (Fig. 3). Although a decrease in hydrolysis by the enzymes in the presence of heparin of the synthetic substrates, MeO-Suc-Ala-Ala-Pro-Phe-pNan and MeO-Suc-Ala-Ala-Pro-Val-pNan, was respectively observed, inhibitions do not appear to follow the slow tight-binding mechanism as occurs to rBbCI-His_(6)_.

### Reduction of thrombus weight after treatment with heparin, rBbCI-His_(6)_, or heparin plus rBbCI-His_(6)_

**Figure 4:**
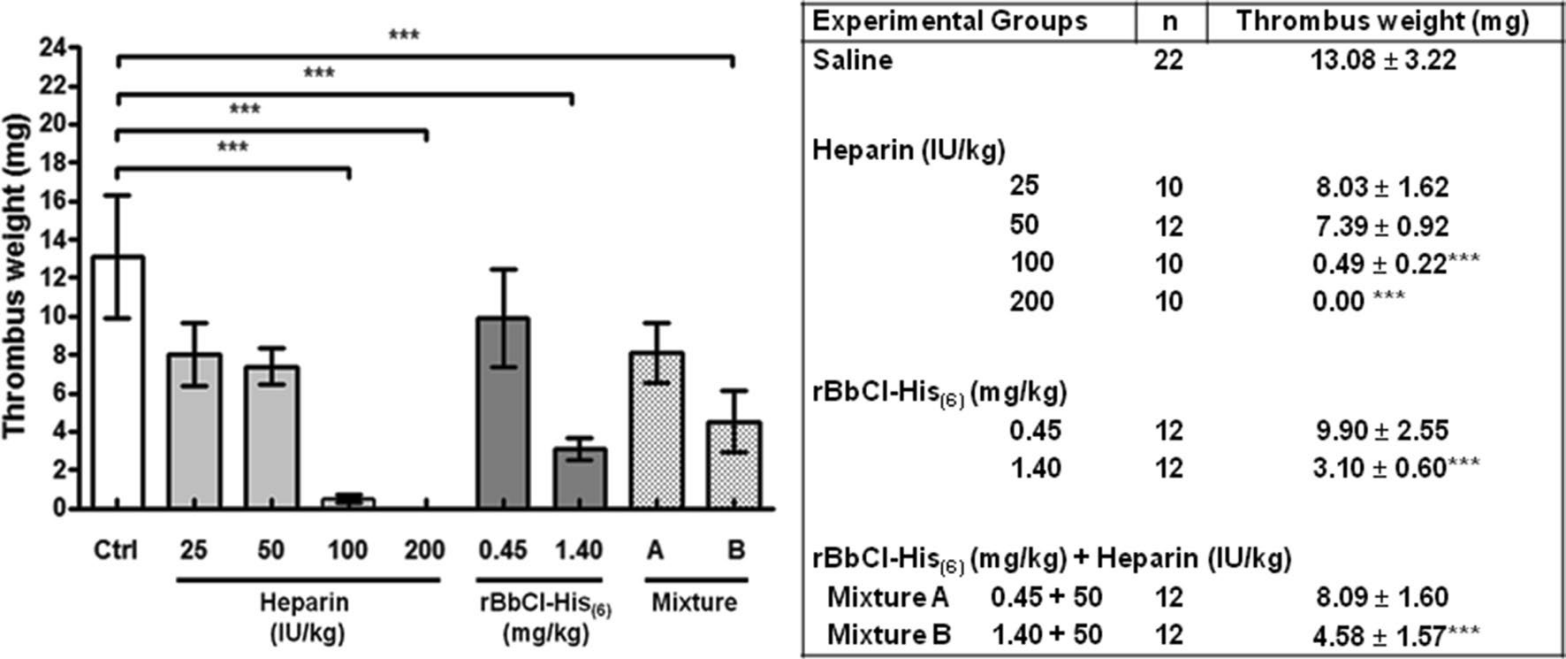
Effect of heparin, rBbCI-His_(6)_, and heparin plus rBbCI-His_(6)_ on thrombus weight. Rats were treated intravenously with saline (control), heparin, inhibitors, or heparin plus rBbCI-His_(6)_ for 30 min before VC ligature. The thrombus was removed and weighted 4 h after the ligature (“ex-vivo” group). Values are expressed as mean ± s.d.. *p˂0.05, **p˂0.01, and ***p˂0.001 versus the corresponding values in the saline group.

All tested substances reduced thrombus weight when compared to the control group (Fig. 4). Treatment with heparin at the doses of 25 or 50 IU/kg reduced thrombus weight by 38 and 43%, respectively. Heparin at doses of 100 or 200 IU/kg reduced the thrombus weight by 96% and 100% (absence of thrombus), respectively, showing significant values. Both doses of rBbCI-His_(6)_ (0:45 or 1.4 mg/kg) reduced thrombus weight in 24 and 76%, respectively. Nevertheless, only a dose of 1.4 mg/kg was statistically significant. The association treatment mixture A and B reduced thrombus weight by 38% and 65%, respectively; however, only mixture B showed a significant value. It is worth mentioning that this association showed a smaller effect on the reduction of thrombus weight when compared to the treatment with the inhibitor alone at a dose of 1.4 mg/kg.

### Serum levels of cytokines in rBbCI-His_(6)_, heparin, and heparin plus rBbCI-His_(6)_ treated groups

**Figure 5:**
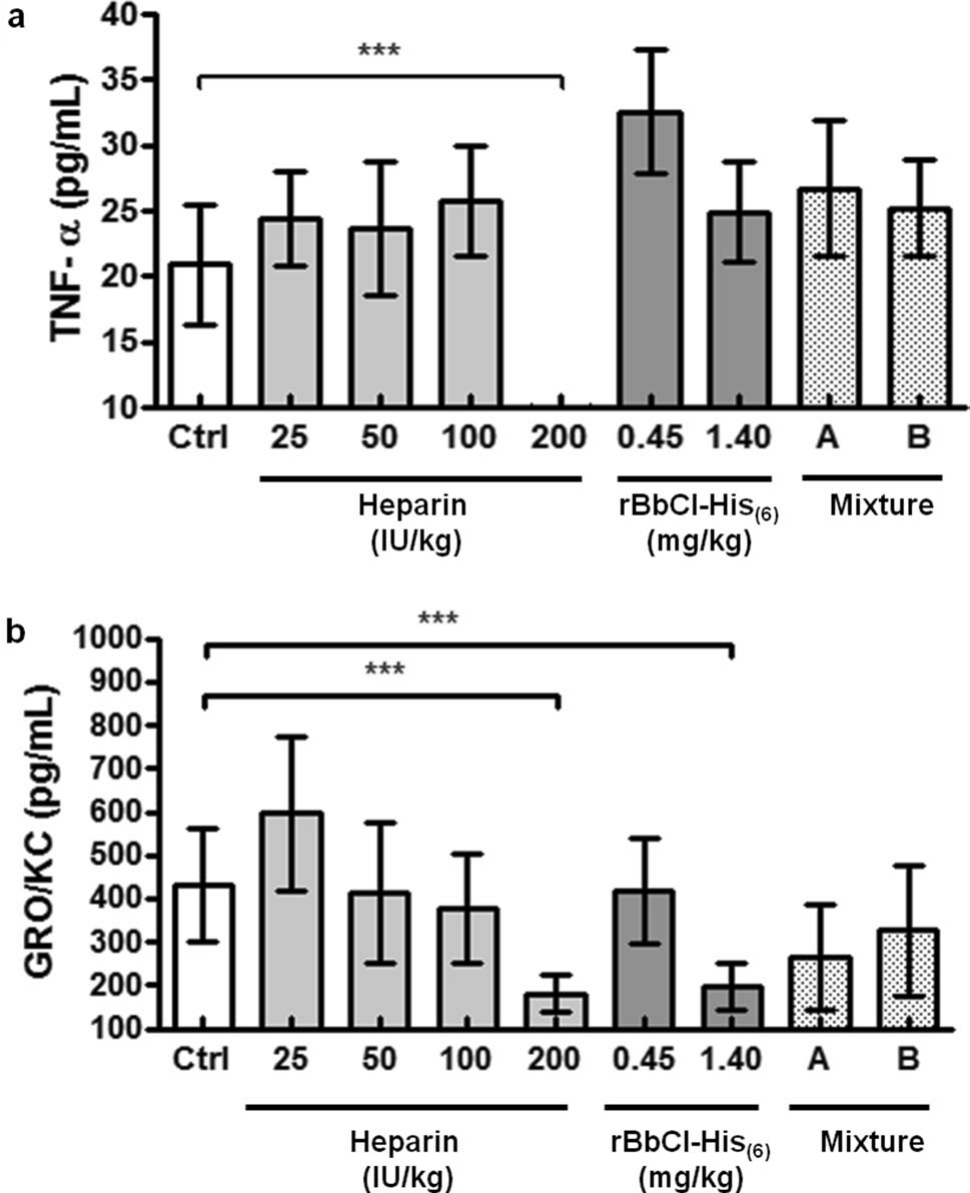
Effect of rBbCI-His_(6)_ associated or not with heparin on serum cytokines levels (TNF-alpha and GRO/KC). Serum samples were collected 4 h after thrombosis induction in rats treated intravenously with saline, inhibitors, or heparin plus rBbCI-His_(6)_ 30 min before ligature (“ex-vivo” group). The concentrations of cytokines were determined in the Milliplex system. Values are expressed as mean ± s.d.. ***p˂0.001 versus the corresponding values in the saline group.

Cytokines (TNF-alpha, IL-1α, IL-1β, and IL-6) and chemokines (GRO/KC, MCP-1, and MIP-1α) were quantified in the serum of rats in the experimental groups 4 h after VC ligature. In the experimental conditions, TNF-alpha and GRO/KC showed the only decrease in serum levels observed, considering the conditions of time and collection procedure (Fig. 5).

The treatment with heparin (200 IU/kg) was the only one showing a significant reduction in serum TNF-α (73%) compared to the saline group (Fig. 5a). Both treatments, with heparin (200 IU/kg) or rBbCI-His_(6)_ (1.4 mg/kg), were shown to have a similar and significant decrease in the serum concentration of GRO/KC (58 and 54%, respectively) compared to the saline group (Fig. 5b). However, the combined treatment with rBbCI-His_(6)_ plus heparin (mixture A and B) did not provide additive or synergistic effects. In the sham surgery group and substances control group, the serum levels of the studied cytokines were not different from those in the saline group (data not shown).

## Discussion

In the present study, the recombinant form of BbCI was used in an experimental model of venous thrombosis, exploring the advantage that it can be produced easily, pure, and in large quantities. Furthermore, the histidine tail in rBbCI-His_(6)_ did not impair its inhibitory activity on HNE. Conversely, the ability to inhibit CG in relation to the native protein, or the recombinant form depleted from the histidine tail, was enhanced, which reduces the cost and optimizes the method for protein production on a large scale. The integrity of the preparation, verified by mass spectrometry, ruled out a possible degradation of the inhibitor by thrombin that could cause the loss of inhibition efficacy. The lack of activity on PR-3 may be related to the additional sequence present in recombinant forms^29^, which may have interfered in association with this enzyme.

The capability of rBbCI-His_(6)_ to reduce thrombus development in an experimental model of VC ligature in rats was comparable to that of heparin, the most frequent antithrombotic used drug^33^. However, rBbCI-His_(6)_ distinguishes it from heparin because it did not alter the coagulation parameters or prolonged the bleeding time in the tail. With heparin at doses of 100 or 200 IU/kg, although there is no thrombus formation an intense anticoagulant effect at the time of vena cava ligature is noticed. Four hours after ligature, the values of aPTT in the rat are not statistically different from the saline group values. These values are possible due to the route of administration, the doses used, and heparin clearance^34,35^ however, this does not mean the end of its antithrombotic effect as no thrombus was present at this time.

More and more studies are correlating the formation of thrombi with inflammation, both in the case of arterial and venous thrombosis^16,36–38^.

It is reported that heparin, in addition to its anticoagulant activity, also has an anti-inflammatory "in vitro” and "in vivo" action due to the inhibitory action on CG and HNE^39–41^, suggesting that these enzymes play an essential role in the establishment of thrombosis^42^. This statement was also confirmed by our results because at the time of VC ligature in the animal group that received low doses of heparin (25 and 50 IU/kg), the thrombus mass was approximately 40% reduced without alteration in the aPTT and BT. At high doses of heparin (100 and 200 IU/kg), although the thrombus formation was completely prevented at these doses, the aPTT was therapeutically altered with prolongation in BT, increasing the hemorrhagic risk. On the other hand, the rBbCI-His_(6)_ recombinant inhibitor from plant origin reduced the thrombus development without altering the coagulation parameters and BT. Although rBbCI-His_(6)_ and heparin act on the same inflammatory enzymes (CG and HNE), unlike heparin, the inhibitor does not block coagulation enzymes such as factors IIa and Xa.

Moreover, the efficacy of rBbCl-His_(6)_ in blocking the action of these neutrophil enzymes is approximately 1000-fold higher than that of heparin, which leads to the conclusion that the antithrombotic properties of rBbCl-His_(6)_ may be a consequence of its anti-inflammatory properties attributed mainly to HNE and CG blocking. The rBbCI-His_(6)_ inhibitor also has an advantage over other elastase and CG inhibitors, as it is resistant to inactivation by oxidant compounds and may block these extracellularly exposed enzymes under inflammatory conditions^27–29^. Due to this ability to resist oxidation, possibly rBbCI-His_(6)_ may contribute to reducing the activity of these enzymes present in the NET structure during the thrombus propagation phase^43^. This anti-inflammatory activity helps the prevention of local coagulation activation and thrombus formation. Overall, the HNE and CG inhibition by rBbCI-His_(6)_ may reduce the activation of endothelial cells and/or monocytes, reducing the local release of tissue factors by these cells^44–46^. Besides, blocking HNE, the cleavage of AT by this enzyme may decrease, consequently thrombin activity is better controlled by the endogenous inhibitor^47,48^. Furthermore, inhibiting CG may prevent the activation of factors V, VIII, and FX^49–52^, reducing the procoagulant state.

The neutrophils serine proteases regulate different phases in the sequential mechanisms of cell activation, adhesion, and migration, which are mediated by the expression of adhesion molecules and cytokines^53,54^ In this respect, the native BbCl protein locally and systemically reduced the CINC-1, chemokine that belongs to the interleukin-8 family expressed in rats, specific form by approximately 60%, blocking neutrophil adhesion and migration towards the activated venular endothelium^28^. A comparable behavior is attributed to rBbCI-His_(6)_.

A reduction of IL-8 levels may lead to decreased neutrophil attraction and degranulation^55^, as well as a reduction in the pro-coagulant action on the endothelium^56^ and thus, a reduction of this cytokine by rBbCI-His_(6)_ also contributes to its antithrombotic action by reducing coagulation, whereas IL-8 induces the release of tissue factor by monocytes^57^.

Conversely, the possibility of small foci of tissue injury caused by the ligament trauma itself and the distension of the adjacent vascular wall, which could lead to the release of tissue factor and adhesion and platelet activation, should also be considered in this complex system^58,59^. In this situation, the inhibition of CG and HNE by rBbCI-His_(6)_ may interfere with the contribution of platelets for the development of thrombi by inhibiting the cleavage of α2β3 integrin by HNE hindering the binding of fibrinogen to integrin^60,61^. Additionally, the inhibited CG prevents the cleavage of PAR4, and activation of local platelet aggregation is will be reduced^62^. To increase the antithrombotic effect with less risk of bleeding, we attempted to associate the inhibitor (0.45 and 1.4 mg/kg) with the lowest dose of heparin (50 IU/kg) but this associated treatment did not show any advantage over the use of the inhibitor alone, as it did not modify the anticoagulant effect and the BT of heparin at the time of VC ligature nor the thrombus on weight. At the moment we do not know how to explain this result, it is possible that “in vivo” the administration of heparin and rBbCI-His_(6)_ found an environment that reduced the activity of the inhibitor; however further investigation is required. On the other hand, rBbCI-His_(6)_ alone was able to reduce the development of thrombi without bleeding and may in the future be a possible alternative for prophylaxis and treatment of venous thrombosis.

A limitation of our study is that, in addition to the determination of cytokines, we could have investigated other inflammatory markers that could aid to better characterize the anti-inflammatory action of rBbCI-His_(6)_ in venous thrombosis, which we intend to include in complementary studies.

In conclusion, our results demonstrate that the inhibition of neutrophil proteases, HNE and CG, as well as the reduction of the GRO/KC level by rBbCI-His_(6)_, reduces thrombus formation without changing the parameters of coagulation and bleeding time, reinforcing the role of inflammation in the development of venous thrombosis and suggesting the possibility of a less hemorrhagic therapy. The properties exhibited by rBbCI-His_(6)_ so far encourage us to continue the studies not only to address other relevant issues in this work, but also to investigate the reduction of IL-8 and elastase in the structure of the thrombus, fibrinolysis, and resolution, contributing to the understanding of the phases involved in thrombotic events.

## Supplementary Information

Supplementary Figure S1.
