# Supplementary material for: The recombinant plant Bauhinia bauhinioides elastase inhibitor reduces rat thrombus without alterations in hemostatic parameters
Source: Sci Rep. 2021 Jun 29;11:13475. doi: 10.1038/s41598-021-92745-4 (PMC8241853; doi:10.1038/s41598-021-92745-4)
Supplement: Supplementary file 1 — Supplementary Figure S1. [file 41598_2021_92745_MOESM1_ESM.docx]

**The recombinant plant *Bauhinia bauhinioides* elastase inhibitor reduces rat thrombus without alterations in hemostatic parameters**

Cleide de Oliveira^1^, Mayara Vioto Valois^1^, Tatiana Fontes Ottaiano^1^, Antonio Miranda^2^, Daiane Hansen^1^, Misako Uemura Sampaio^1^, Maria Luiza Vilela Oliva^1^***** and Francisco Humberto de Abreu Maffei^3^

Departamento de ^1^Bioquímica e ^2^Biofísica, Universidade Federal de São Paulo, 04044-020, São Paulo, SP, Brazil;

^3^Departamento de Cirurgia e Ortopedia, Universidade Estadual Paulista, 18618-970, Botucatu, SP, Brazil.

**Corresponding Author**: *****Maria Luiza Vilela Oliva, Departamento de Bioquímica, Universidade Federal de São Paulo, Rua Três de Maio, 100, 04044-020, São Paulo, Brazil. Phone: +55 11 55764445. e-mail: [mlvoliva@unifesp.br](about:blank)

**Supplementary Material**

**
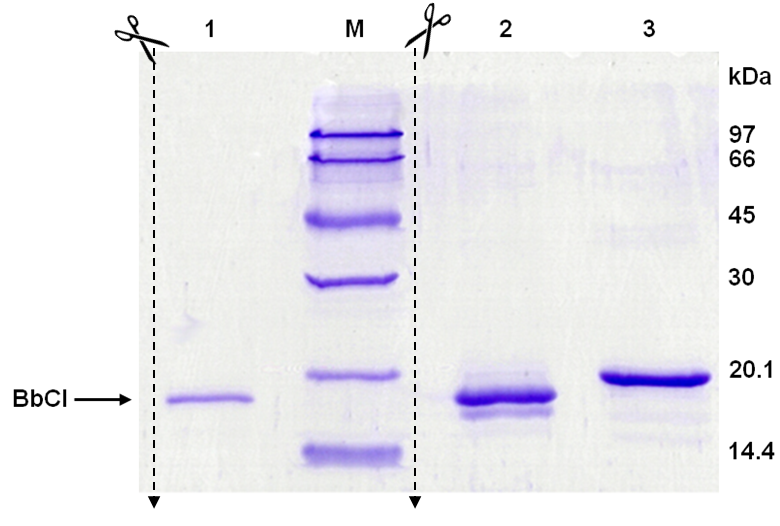
**

**Figure 1. (a) Original image of BbCI electrophoresis. SDS-PAGE (15%) stained with Coomassie-blue.** **(a)**Protease inhibitors after reverse phase chromatography and under reducing conditions. **1)** BbCI, **2)** *Bauhinia rufa* elastase inhibitor (type I), **3)** *Bauhinia rufa* elastase inhibitor (type II), **M)** molecular mass standard, rabbit phosphorylase b 97.0 kDa, bovine serum albumin 66.0 kDa, egg albumin 45.0 kDa, bovine carbonic anhydrase 30.0 kDa, soybean trypsin inhibitor 20.1 kDa, and bovine alpha-lactalbumin 14.4 kDa.


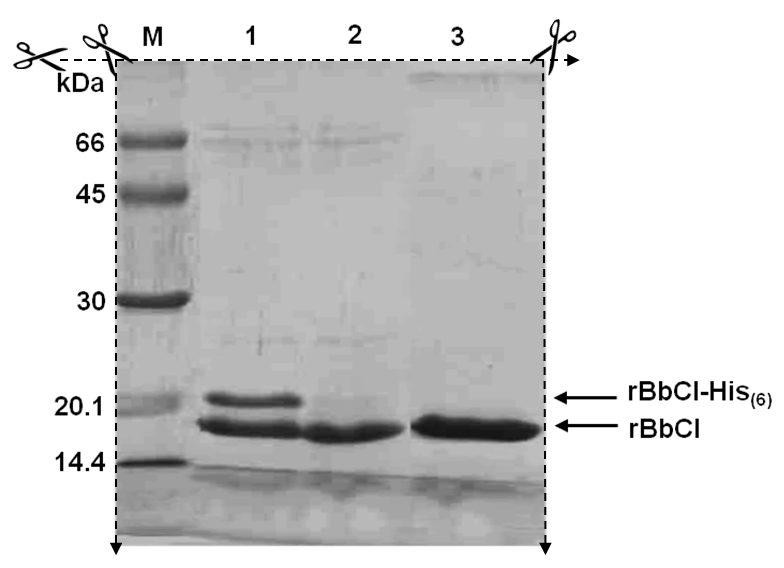


**Figure 1. (c) Original image of the electrophoretic migration of rBbCI and rBbCI-His_(6)._** The gel (SDS-PAGE (15%)) was scanned (HP Photosmart C4280) to obtain the image which is shown in black and white. **M)** Molecular mass standard, bovine serum albumin 66.0 kDa, egg albumin 45.0 kDa, bovine carbonic anhydrase 30.0 kDa, soybean trypsin inhibitor 20.1 kDa, and bovine alpha-lactalbumin 14.4 kDa. The recombinant protein was subjected to cleavage with 0.5 IU of thrombin at 18 ° C for time intervals (1h) or (4h) 1) and 2), respectively. 3) rBbCl after thrombin cleavage (4h) and molecular exclusion chromatography on a Superdex 75 column equilibrated with 0.1 M Tris-HCl buffer (pH 8.0).

**
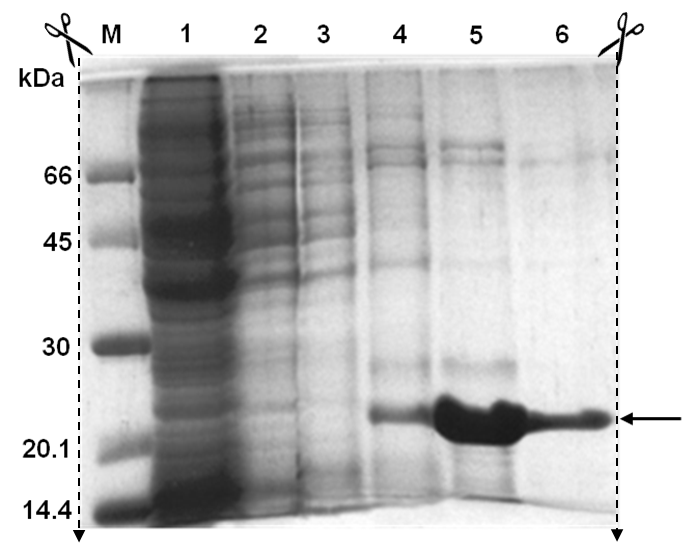
**

**SDS-PAGE (15%)** **electrophoresis of rBbCI-His_(6)_ confirming the inhibitor purification and the gel migration profile. M)** Molecular mass standard, bovine serum albumin 66.0 kDa, egg albumin 45.0 kDa, bovine carbonic anhydrase 30.0 kDa, soybean trypsin inhibitor 20.1 kDa, and bovine alpha-lactalbumin 14.4 kDa. **1)** crude extract (soluble fraction of pETBbCI expression in *E. coli* BL21 (DE3)); **2)** proteins eluted with 100 mM Tris-HCl pH 8.0 and 150 mM NaCl; **3** to **6)** proteins eluted with 100 mM Tris-HCl pH 8.0 buffer and 150 mM NaCl containing 10 to 500 mM imidazole. The arrow indicates rBbCI-His_(6)_ eluted from the affinity chromatography in Ni-NTA (nickel-nitrilotriacetic acid) resin.
